# Highly purified mussel adhesive protein to secure biosafety for *in vivo* applications

**DOI:** 10.1186/1475-2859-13-52

**Published:** 2014-04-11

**Authors:** Bong-Hyuk Choi, Hogyun Cheong, Yun Kee Jo, So Yeong Bahn, Jeong Hyun Seo, Hyung Joon Cha

**Affiliations:** 1 Department of Chemical Engineering, Pohang University of Science and Technology, Pohang 790-784, Korea; 2 School of Interdisciplinary Bioscience and Bioengineering, Pohang University of Science and Technology, Pohang 790-784, Korea; 3 School of Chemical Engineering, Yeungnam University, Gyeongsan 712-749, Korea

**Keywords:** Mussel adhesive protein, Gram-negative *Escherichia coli*, High resolution purification, Biosafety, Lipopolysaccharide, Endotoxin, *In vivo* standard

## Abstract

**Background:**

Unique adhesive and biocompatibility properties of mussel adhesive proteins (MAPs) are known for their great potential in many tissue engineering and biomedical applications. Previously, it was successfully demonstrated that redesigned hybrid type MAP, fp-151, mass-produced in Gram-negative bacterium *Escherichia coli*, could be utilized as a promising adhesive biomaterial. However, purification of recombinant fp-151 has been unsatisfactory due to its adhesive nature and polarity which make separation of contaminants (especially, lipopolysaccharide, a toxic Gram-negative cell membrane component) very difficult.

**Results:**

In the present work, we devised a high resolution purification approach to secure safety standards of recombinant fp-151 for the successful use in *in vivo* applications. Undesirable impurities were remarkably eliminated as going through sequential steps including treatment with multivalent ion and chelating agent for cell membrane washing, mechanical cell disruption, non-ionic surfactant treatment for isolated inclusion body washing, acid extraction of washed inclusion body, and ion exchange chromatography purification of acid extracted sample. Through various analyses, such as high performance liquid chromatographic purity assay, limulus amoebocyte lysate endotoxin assay, and *in vitro* mouse macrophage cell tests on inflammation, viability, cytotoxicity, and apoptosis, we confirmed the biological safety of bacterial-derived purified recombinant fp-151.

**Conclusions:**

Through this purification design, recombinant fp-151 achieved 99.90% protein purity and 99.91% endotoxin reduction that nearly no inflammation response was observed in *in vitro* experiments. Thus, the highly purified recombinant MAP would be successfully used as a safety-secured *in vivo* bioadhesive for tissue engineering and biomedical applications.

## Background

Mussels use unique protein-based bioadhesives that can maintain strong adhesiveness even in the aquatic environment to survive in the ocean [1,2]. Mussel adhesive proteins (MAPs) are also known for displaying excellent biocompatibility and biodegradability [3-5]. These unique properties make MAPs promising and valuable biomaterials that can be utilized in different tissue engineering and medical applications [6,7]. Since extracting natural adhesive proteins from mussels is a labour intensive and cost ineffective process, mass-production of recombinant MAPs has been intensively attempted for practical use of MAP [1,8-12]. Previously, recombinant hybrid type MAP fp-151, composed of six repeats of type 1 protein (fp-1) decapeptide at both N- and C-termini of type 5 protein (fp-5), was successfully designed and obtained in *Escherichia coli* system with high production and purification yields [13].

Even though the establishment of recombinant fp-151 production has overcome the limitation in quantity, this system still requires much improvement on its purity and safety perspectives due to the undesirable impurities caused by Gram-negative bacterium *E. coli* during cell disruption and protein purification process. Due to adhesive nature and positive polarity, purification of recombinant MAPs at high purity suitable for *in vivo* applications has been a difficult task. Formally, recombinant fp-151 expressed in cytoplasm has been recovered using simple acid extraction method from surfactant Triton X-100-treated inclusion body [13]. However, this method cannot fully remove impurities that MAP purified in this manner does not show satisfactory purity nor safety *in vivo* situations. In addition, those impurities are considered to contain large amount of lipopolysaccharides (LPS) which are macro molecules consisting of a lipid and a polysaccharide linked by a covalent bond and are found in the outer membrane of Gram-negative bacteria. LPS causes serious endotoxin-related immune response problems in higher living organisms, such as fever, shock and even death [14-16]. Thus, developing an effective and reliable purification process to ensure high purity and biological safety of recombinant MAP became an important assignment to exploit it for practical *in vivo* biomedical applications.

In the present work, a high resolution purification process was proposed to enhance purity and ensure biological safety of *E. coli*-derived recombinant fp-151. This purification process was composed of three strategies targeting different steps of protein recovery: 1) multivalent ion and chelating agent treatment of whole cells to reduce cell membrane components, 2) non-ionic surfactant washing of isolated inclusion body to reduce undesirable contaminants, and 3) final ion exchange chromatographic recovery. The efficacy of this purification process was assessed in many aspects of purity and safety using various analyses including protein quantification, endotoxin assay, and *in vitro* macrophage cell tests.

## Results and discussions

**Figure 1:**
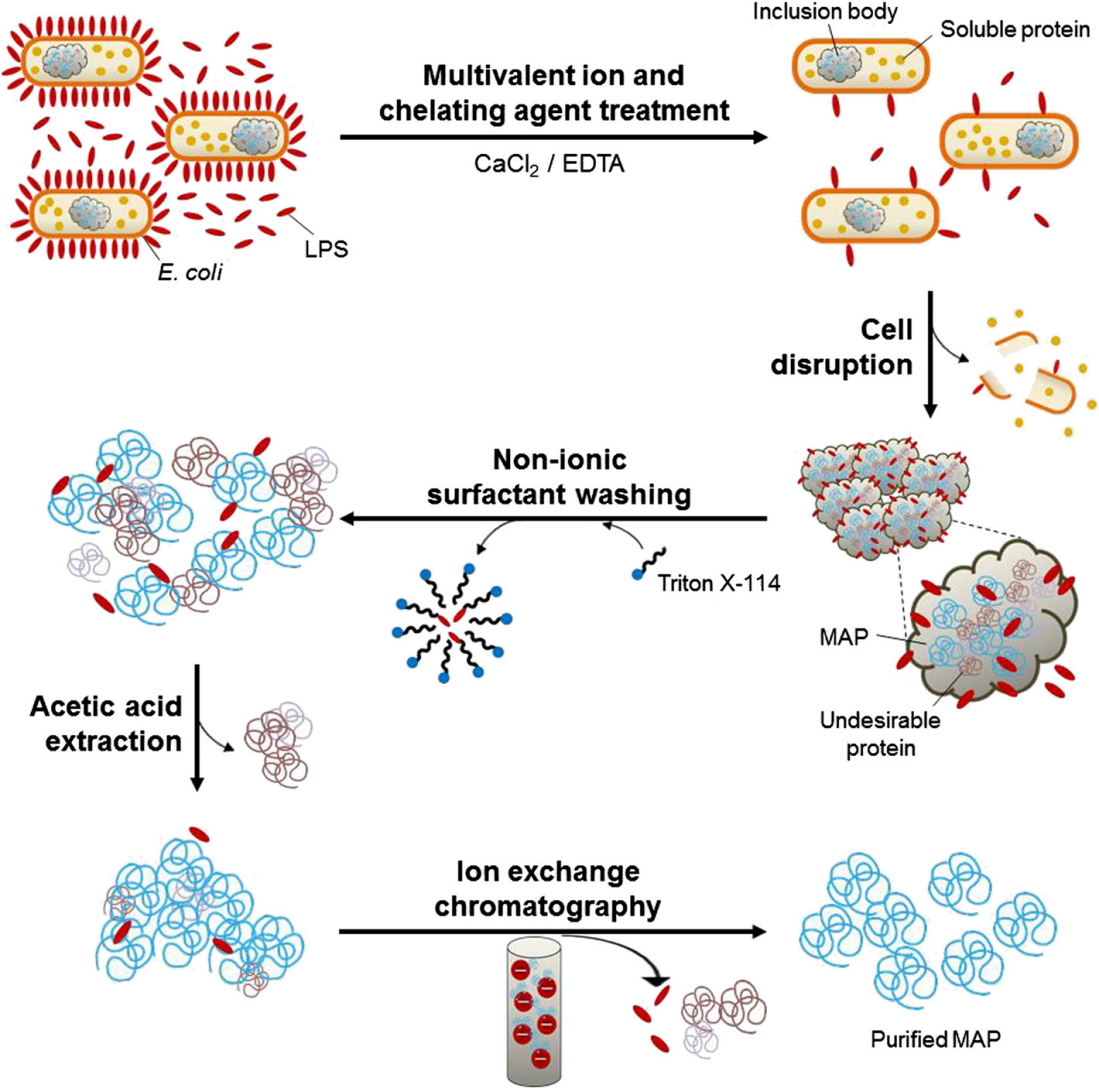
**Schematic representation of high resolution purification process for cytoplasmic expressed recombinant MAP in Gram-negative *E. coli*.**

We designed a purification process composed of three strategic steps in series to satisfy purity and biological safety of recombinant MAP fp-151 for potential use in *in vivo* experiments through the effective removing of impurities such as contaminated proteins and LPS; 1) inclusion body isolation after disruption of CaCl_2_/ethylenediaminetetraacetic acid (EDTA)-treated harvested cells, 2) acetic acid extraction after double washing of isolated inclusion body with Triton X-114, and 3) ion exchange chromatography of acid-extracted supernatant (Figure 1). The purity and endotoxin levels of purified recombinant MAP were taken into consideration to verify the efficacy of purification because they are the most important safety parameters.

### High resolution purification of recombinant MAP

There are many strategies to improve purity and safety that are applicable for protein purification [17,18]. In the case of bacterial cytoplasmic expression system such as Gram-negative bacterium *E. coli*, cell membrane should be disrupted to obtain target heterologous proteins. As cell membrane is shattered during cell lysis, a lot of undesirable molecules such as proteins, lipids, and carbohydrates are released into cell lysate. The main source of impurities during protein purification is LPS which is found in the outer membrane of gram-negative bacteria and constitutes a large portion of cell membrane contents [14]. Thus, effectively removing LPS for *E. coli* system is thought as a key to ensure the purity and safety of a final product [16,18-23]. Our previous MAP purification method was based on simple acetic acid extraction after single Triton X-100 washing of isolated inclusion body [13]. Thus, purity and biosafety of purified MAP were not guaranteed for *in vivo* standards. Because adhesive and positively charged nature of MAPs, they have high chance to interact with many types of impurities, especially negatively charged cell components such as LPS.

To remove cell membrane components from protein solutions, differential centrifugation technique, such as sucrose gradient centrifugation, is commonly used [24]. However, this is not suitable for the large-scale purification because it is a very time consuming method that requires more than 10–20 h of ultracentrifugation. Another interesting method is membrane washing with multivalent ions such as CaCl_2_ and chelating agents such as EDTA, which directly treats living cells to clean up cell membrane components [25-27]. This method is quite simple and fast compared to sucrose gradient centrifugation. Thus, it is considered to be suitable for large-scale purification of recombinant MAP. Another big source of impurities resides in the inclusion body which contains some unwanted or misfolded proteins as well as the desired protein. Moreover, inclusion body is inevitably contaminated by LPS after cell lysis, so inclusion body should be also cleaned up efficiently. For inclusion body washing, non-ionic surfactants are common choices because they are strong enough to separate proteins while weak enough not to harm the protein structure [28]. Especially, Triton X-100 and Triton X-114 are popular non-ionic surfactants known for effectively separating LPS from proteins [23,28-30].

**Figure 2:**
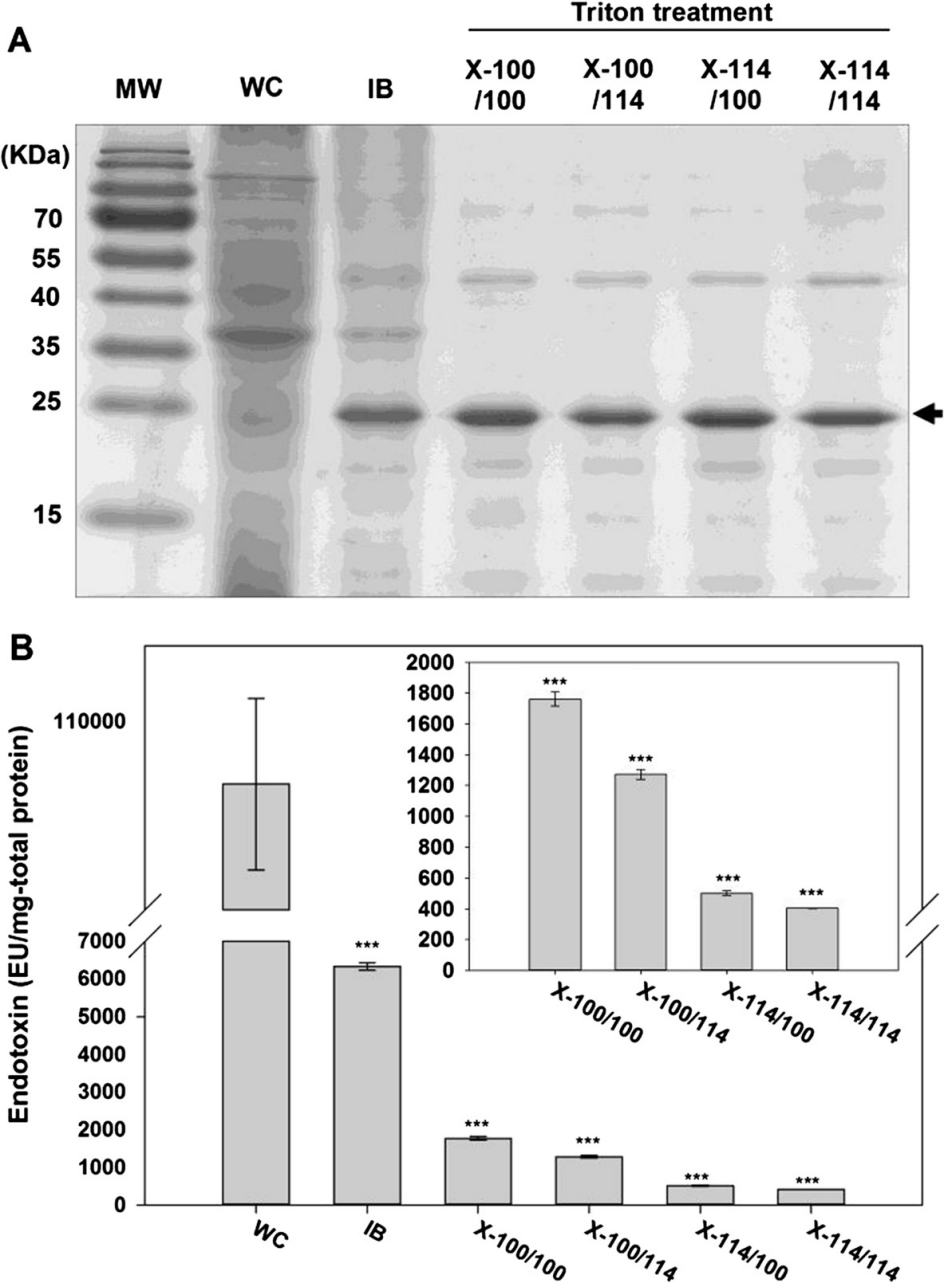
**(A) SDS-PAGE analysis and (B) endotoxin level determination of recombinant MAP samples according to each purification step before ion exchange chromatography.** The data represent mean +/− standard deviation and were analyzed 2-tails student t-test (*p < 0.05, **p < 0.01, ***p < 0.005). Abbreviations: MW, molecular weight marker; WC, whole cells; IB, isolated inclusion body containing MAP after disruption of CaCl_2_/EDTA-treated whole cells; X-100/100, acetic acid-extracted MAP after inclusion body washing with Triton X-100 twice; X-100/114, acetic acid-extracted MAP after inclusion body washing with Triton X-100 and Triton X-114 in order; X-114/100, acetic acid-extracted MAP after inclusion body washing with Triton X-114 and Triton X-100 in order; X-114/114, acetic acid-extracted MAP after inclusion body washing with Triton X-114 twice.

As pre-treatment of harvested whole cells, CaCl_2_/EDTA washing was used to remove LPS from the cell wall so that the chance of LPS contamination for MAP during the cell lysis can be reduced (Figure 1). After the cell pre-treatment, somewhat LPS-reduced cells were prepared for cell disruption. Then, non-ionic surfactant treatment was performed for washing the isolated inclusion body containing recombinant MAP (Figure 1). For the surfactant washing, Triton X-100 and Triton X-114 were assessed in combination to establish the best performing condition against LPS. Sodium dodecyl sulfate polyacrylamide gel electrophoresis (SDS-PAGE) analysis showed that recombinant MAP was properly recovered at each step of purification as a major band that appeared at ~23 kDa (Figure 2A). After adjusting the concentration of recombinant MAP from each purification step, endotoxin levels at each step were assessed through limulus amoebocyte lysate (LAL) assay (Figure 2B). We found that CaCl_2_/EDTA treatment reduced endotoxin level of MAP below 1/4 value of crude whole cell lysate (Figure 2B and Table 1). This result strongly suggests that multivalent calcium ions attach to negatively charged LPS residues on the cell membrane and then, EDTA chelation on those calcium ions effectively removes LPS [25-27].

**Table 1:** Yield, endotoxin level, and purity of recombinant MAP in each purification step

**Step**	**Whole cells**	**Inclusion body isolation after disruption of CaCl**_**2**_**/EDTA-treated cells**	**Acetic acid extraction after Triton X-114 double washing of inclusion body**	**Cation exchange chromatography of extracted fraction**
Total weight (mg)	25,500.00	9,600.00	516.00	275.47
Production yield (μg/mL)	7,697.80	7,309.75	2,956.25	1,578.21
Relative production yield (%)	100.00	94.96	38.40	20.50
Endotoxin level (EU/mg-total protein)	108,332.01	6,324.79	403.45	22.33
Relative endotoxin level (%)	100.00	5.84	0.37	0.02
Purity (%)	-	35.30^a)^	89.81^b)^	99.90^b)^

^a)^calculation based on SDS-PAGE analysis.

^b)^calculation based on HPLC analysis.

During the protein recovery step through acetic acid extraction of inclusion bodies washed with non-ionic surfactant (Figure 1), the sample washed twice with Triton X-114 presented the best performance in endotoxin removal among four combinations of Triton X series (inner plot in Figure 2B). Even though Triton X-100 and Triton X-114 are sister chemicals, Triton X-114 has shorter hydrophilic arm than Triton X-100 has; because Triton X-114 has lower hydrophilic-lipophilic balance, it has advantage over Triton X-100 when interacting with heavy lipid chain of LPS [22,23]. Overall, cell pre-treatment using CaCl_2_/EDTA and inclusion body washing with Triton X-114 greatly contributed to reduction of LPS contents in the recombinant MAP along the purification process.

**Figure 3:**
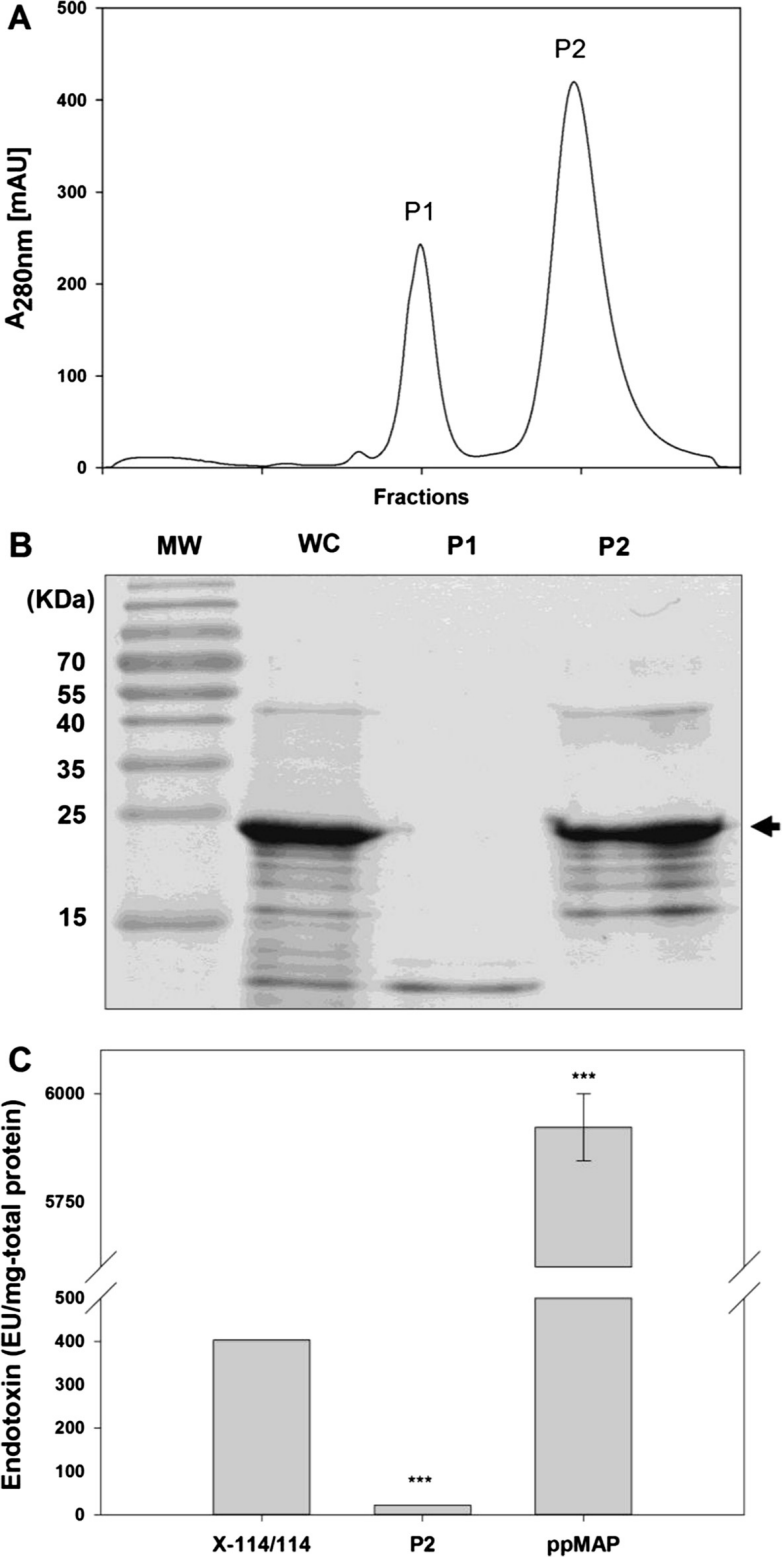
**(A) Ion exchange chromatogram, (B) SDS-PAGE analysis, and (C) endotoxin level determination of recombinant MAP sample after ion exchange chromatography purification.** The data represent mean +/− standard deviation and were analyzed 2-tails student t-test (*p < 0.05, **p < 0.01, ***p < 0.005). Abbreviations: MW, molecular weight marker; WC, whole cells; X-114/114, acetic acid-extracted MAP after inclusion body washing with Triton X-114 twice; P1, first peak fraction from cation exchanger of acetic acid-extracted solution after inclusion body washing with Triton X-114 twice; P2, second peak fraction from cation exchanger of acetic acid-extracted solution after inclusion body washing with Triton X-114 twice; ppMAP, purified MAP based on previous method.

To prepare highly pure and safety secured final protein product, chromatography is a necessary purification method. Several types of preparative chromatography techniques, such as size-exclusion, affinity, and ion exchange, can provide separation of mixtures. Because MAP has high isoelectric point (pI) value about 10 [13], cation exchanger, which has anionic charged group, was used to filter out anionic impurities (Figure 1). We found that protein impurities (P1 of Figure 3A & B) were efficiently removed by salt gradient and the highly cationic MAP was eluted at the last peak (P2 of Figure 3A & B) with greatly improved purity. Several small bands below the main MAP band were considered as degraded forms which are likely to possess similar positive charges with MAP (P2 of Figure 3B). Because the recombinant MAP fp-151 used in this work has highly repetitive sequence [13], the net charge and hydrophobicity of degraded forms would not be different much from MAP. In addition, because negatively charged LPS were also filtered out by cation exchange chromatography, the endotoxin amount was further reduced to nearly 20 EU per 1 mg total protein, which was significantly low value compared to MAP purified by previous method [13] and even to extracted MAP from Triton X-114-washed inclusion body (Figure 3C and Table 1).

### Assessment of purity of recombinant MAP

**Figure 4:**
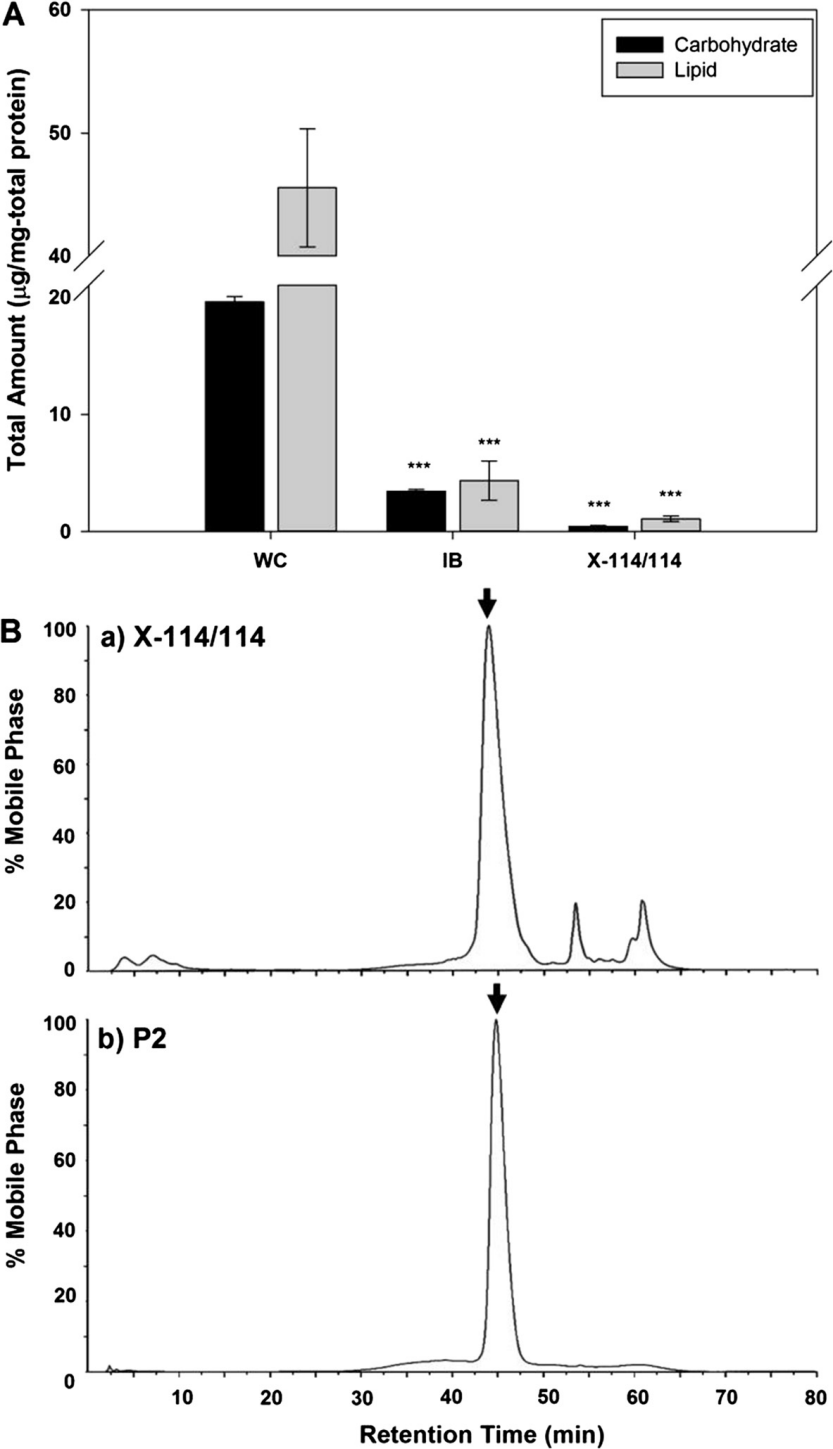
**Assessment of purity of recombinant MAP. (A)** Total carbohydrate/lipid amount determination and **(B)** HPLC chromatograms for protein purity determination. The data represent mean +/− standard deviation and were analyzed 2-tails student t-test (*p < 0.05, **p < 0.01, ***p < 0.005). Abbreviations: WC, whole cells; IB, isolated inclusion body containing MAP after disruption of CaCl_2_/EDTA-treated whole cells; X-114/114, acetic acid-extracted MAP after inclusion body washing with Triton X-114 twice. P2, second peak fraction from cation exchanger of acetic acid-extracted solution after inclusion body washing with Triton X-114 twice.

Just as reducing endotoxin level to secure safety, improving purity is another purpose of this purification process. A purity of recombinant MAP was explored with total carbohydrate/lipid amount determination and high performance liquid chromatography (HPLC) analysis. The total carbohydrate/lipid amount determination is meaningful because it can reveal the amount of cellular components from *E. coli* left in MAP after purification step. We found that cell pre-treatment with CaCl_2_/EDTA cut the total quantities of carbohydrates and lipids to about 1/6 and 1/11 of whole cell lysate, respectively (Figure 4A). In addition, the quantities of carbohydrates and lipids dropped below 1 μg per 1 mg total protein through washing twice with Triton X-114. Thus, these two strategic treatments were proven to be useful in improving the purity of recombinant MAP by reducing the quantities of total carbohydrates and lipids, not just in gaining the safety.

Next, to check protein purity, both MAP samples before (washed twice with Triton X-114) and after being purified by ion exchange chromatography were undergone HPLC analysis. While the sample washed twice with Triton X-114 produced a chromatogram which had one major peak and two minor peaks (Figure 4B-a), where those two minor peaks correspond to the impurities (Figure 3B, P1), the major portion from ion exchange chromatography (Figure 3B, P2) demonstrated only one major peak in HPLC chromatogram (Figure 4B-b). This implies that the degraded forms of MAP (minor bands in Figure 3B, P2), which have virtually the similar hydrophobicity, are eluted at the same point. The purity of recombinant MAP sample after being purified by ion exchange chromatography was significantly increased to 99.9% compared to that (89.8%) of the sample only after acid extraction of Triton X-114-treated inclusion body (Table 1). Consequently, the results of HPLC analyses indicate that ion exchange chromatography step eliminated undesired proteins efficiently and improved the purity of recombinant MAP dramatically. Interestingly, this HPLC results can also explain what those minor bands shown together with MAP in P2 of Figure 3B are; because those minor bands were not separately shown on HPLC chromatogram (Figure 4B-b), they can be considered as degraded forms of MAP, which has similar hydrophobicity that they appear at the same elution point with MAP.

### Assessment of biological safety of purified recombinant MAP

**Figure 5:**
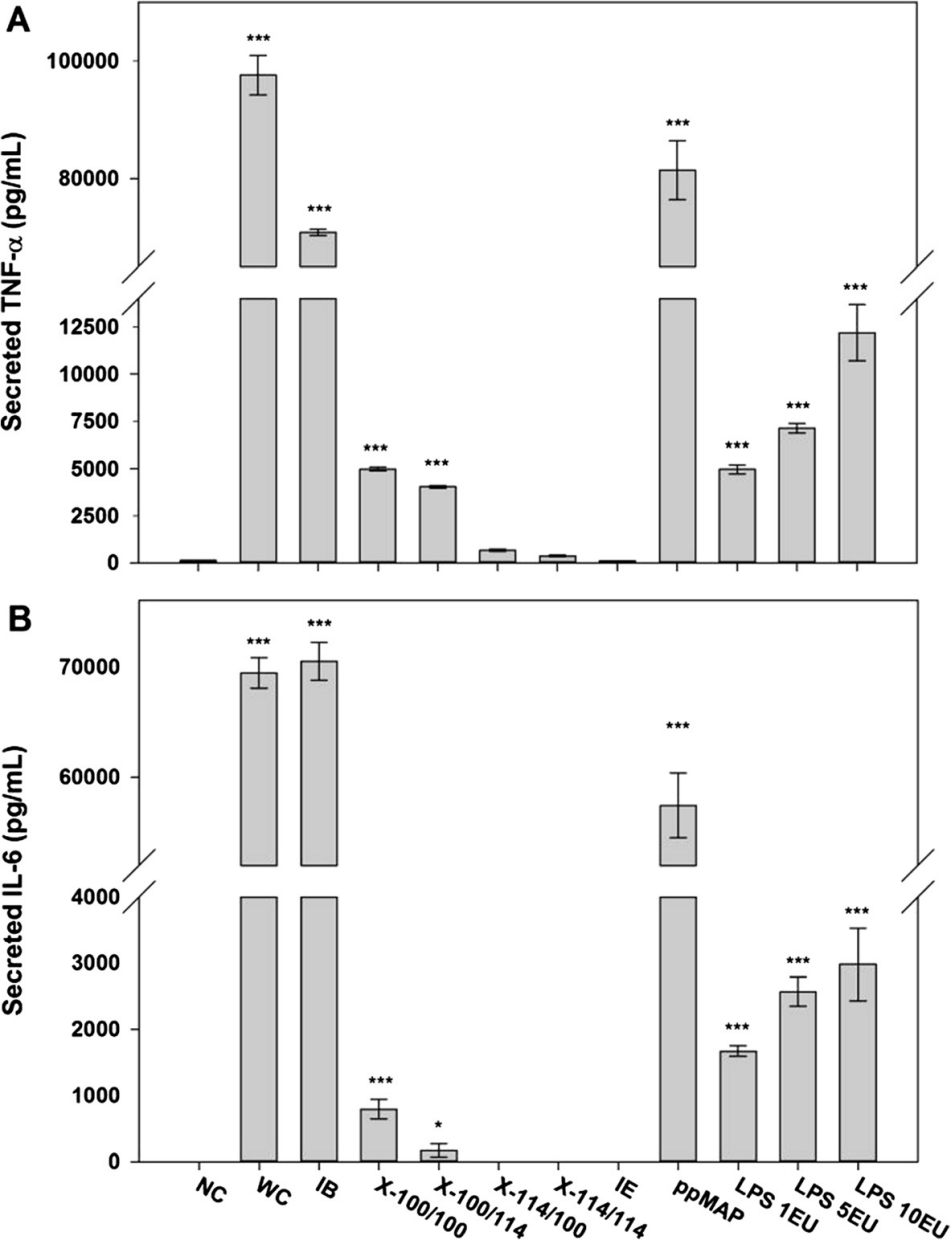
**Assessment of biological safety of purified recombinant MAP using inflammatory response assays on RAW 264.7 macrophage cells. (A)** TNF-α and **(B)** IL-6 mRNA expressions were observed by treating MAP samples at each purification step. The data represent mean +/− standard deviation and were analyzed 2-tails student t-test (*p < 0.05, **p < 0.01, ***p < 0.005). Abbreviations: NC, non-treated negative control; WC, whole cells; IB, isolated inclusion body containing MAP after disruption of CaCl_2_/EDTA-treated whole cells; X-100/100, acetic acid-extracted MAP after inclusion body washing with Triton X-100 twice; X-100/114, acetic acid-extracted MAP after inclusion body washing with Triton X-100 and Triton X-114 in order; X-114/100, acetic acid-extracted MAP after inclusion body washing with Triton X-114 and Triton X-100 in order; X-114/114, acetic acid-extracted MAP after inclusion body washing with Triton X-114 twice; IE: MAP fraction through cation exchange chromatography of acetic acid-extracted solution after inclusion body washing with Triton X-114 twice; ppMAP, purified MAP based on previous method; LPS 1EU, LPS standard with 1 endotoxin unit; LPS 5EU, LPS standard with 5 endotoxin unit; LPS 10EU, LPS standard with 10 endotoxin unit.

**Figure 6:**
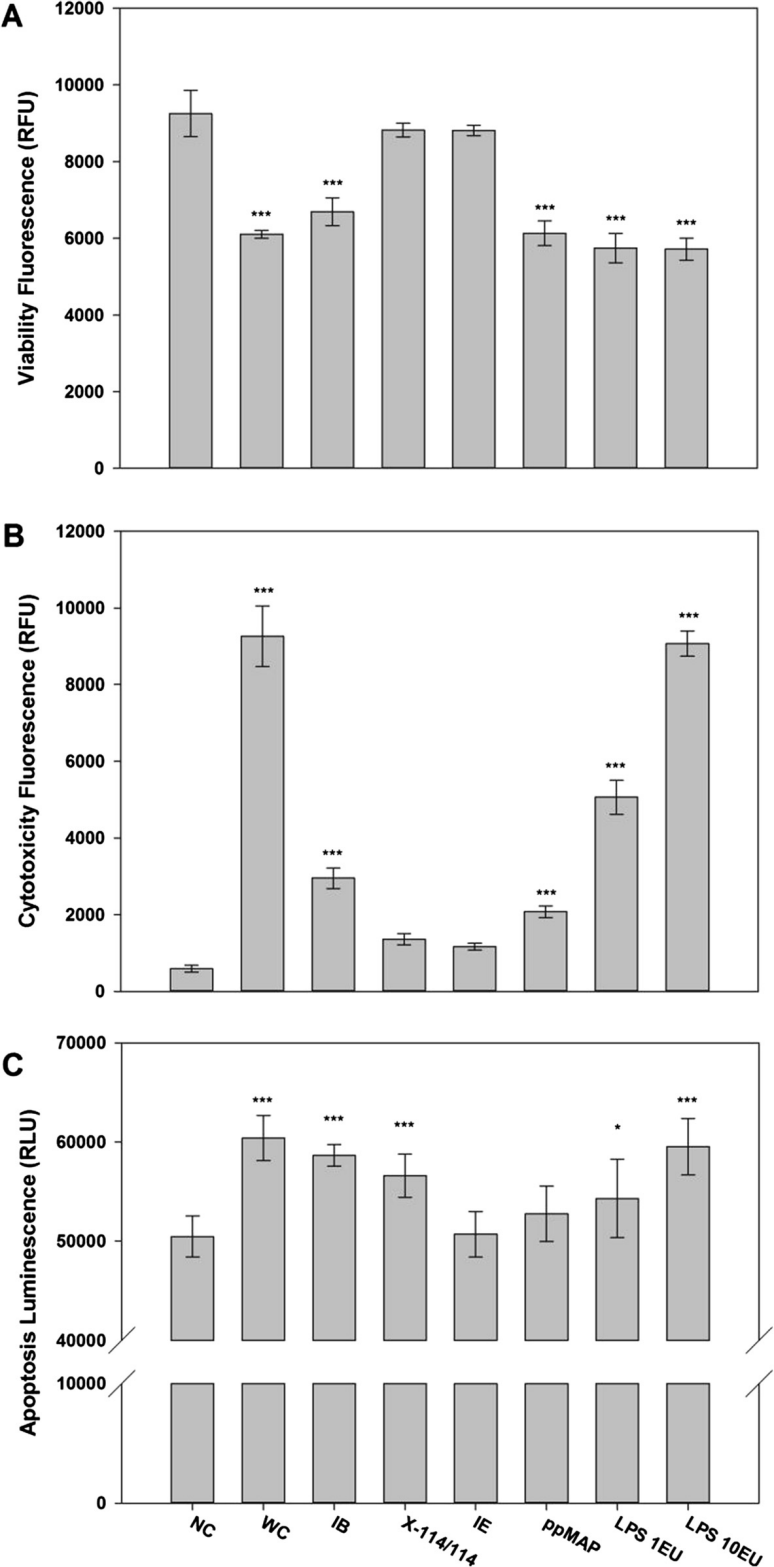
**Assessment of biological safety of purified recombinant MAP using cellular response assays on RAW 264.7 macrophage cells. (A)** Cell viability, **(B)** cytotoxicity, and **(C)** apoptotic cell death were observed by treating MAP samples at each purification step. The data represent mean +/− standard deviation and were analyzed 2-tails student t-test (*p < 0.05, **p < 0.01, ***p < 0.005). Abbreviations: NC, non-treated negative control; WC, whole cells; IB, isolated inclusion body containing MAP after disruption of CaCl_2_/EDTA-treated whole cells; X-114/114, acetic acid-extracted MAP after inclusion body washing with Triton X-114 twice; IE: MAP fraction through cation exchange chromatography of acetic acid-extracted solution; ppMAP, purified MAP based on previous method; LPS 1EU, LPS standard with 1 endotoxin unit; LPS 10EU, LPS standard with 10 endotoxin unit.

Even though most of the impurities were dramatically reduced by the proposed purification process, purified recombinant MAP should meet the biological safety parameters, such as inflammation, viability, cytotoxicity, and apoptosis, for *in vivo* applications. Residual impurities, especially LPS, can cause the innate immune response that expressions of pro-inflammatory cytokines, such as tumour necrosis factor-alpha (TNF-α) and interleukin-6 (IL-6), were assessed using RAW 264.7 macrophage cell line. The expression levels of TNF-α and IL-6 were notably decreased along purification steps and presented lower cytokine expressions than 1 EU LPS standard-treated sample, whereas high levels of TNF-α and IL-6 were observed in recombinant MAP purified by the previous method which was based on simple acetic acid extraction after single Triton X-100 washing of isolated inclusion body [13] (Figure 5). These low activations of inflammatory signal transduction in macrophage cells were ascribed to efficacy of CaCl_2_/EDTA treatment and Triton X-114 washing in LPS reduction. Furthermore, almost no inflammatory cytokines were expressed by final recombinant MAP sample after ion exchange chromatography (Figure 5). This result indicates that the final product of this purification process can be applied to *in vivo* applications without inflammatory response. In addition, other criteria of cellular responses, such as viability, cytotoxicity, and apoptosis, were assessed using macrophage cells to ensure further biosafety of purified recombinant MAP. As results, MAP purified by our purification process showed high viability, low cytotoxicity, and low apoptotic activity compared to positive controls (Figure 6). Taken together, our highly purified recombinant MAP can be successfully used as a safe adhesive biomaterial for *in vivo* applications without triggering cytotoxicity and inflammatory response.

## Conclusions

Here, the establishment of purification process for Gram-negative *E. coli*-derived recombinant MAP, composed of inclusion body isolation after disruption of multivalent ion CaCl_2_/chelating agent EDTA-treated cells, acetic acid extraction after inclusion body washing twice with non-ionic surfactant Triton X-114, and ion exchange chromatography purification of acid-extracted solution with cation exchanger, was achieved to secure high purity and biological safety standards. The purity and safety parameters were confirmed by various assays to prove the efficacy of the proposed purification process. LAL assays revealed that the endotoxins were effectively eliminated; HPLC assay and total carbohydrate-lipid amount determination presented notably improved purity; various *in vitro* macrophage cell tests on inflammatory response and cellular responses such as cell viability, cytotoxicity, and apoptosis confirmed the biological safety of final purified product. This highly purified recombinant MAP with secured biological safety can be successfully used as a promising bioadhesive for *in vivo* tissue engineering and biomedical applications.

## Methods

### Expression of recombinant MAP in *E. coli*

Recombinant fp-151 was produced in *E. coli* as described previously [13]. In brief, transformed *E. coli* BL21 (DE3) cells were cultured in 5 L Luria-Bertani (LB) medium supplemented with 50 μg/mL ampicillin (Sigma, St. Louis, MO, USA) using a 10 L bioreactor (KoBiotech, Incheon, Korea) at 37°C and 300 rpm. At optical density 600 nm (OD_600_) of 0.4–0.6, 1 mM isopropyl-β-D-thiogalactopyranoside (IPTG; Sigma) was added to the broth to induce protein expression, and further cultured for 8 h at 37°C and 300 rpm. After harvesting by centrifugation (Hanil Science Industrial, Incheon, Korea) of culture broth at 9,000 × g for 10 min at 4°C, cell pellets were stored at −80°C for further analysis.

### Inclusion body isolation after disruption of CaCl_2_/EDTA-treated whole cells

Harvested cell pellets containing recombinant fp-151 were washed (resuspended and pelleted) with 15 mL of each washing solution per gram wet weight, in order of 100 mM Tris–HCl (pH 8.0), 5 mM CaCl_2_ in 100 mM Tris–HCl (pH 8.0), and 10 mM EDTA (Sigma) in 100 mM Tris–HCl (pH 8.0). This washing procedure was repeated three times. In the final cycle of washing steps, cell pellets were resuspended in 15 mL lysis buffer (10 mM Tris–HCl and 100 mM sodium phosphate; pH 8.0) per gram wet weight. Cells were lysed with constant cell-disruption system (Constant Systems, Northants, England) at 20 kpsi. Cell lysates were centrifuged at 9,000 × g for 20 min at 4°C, and cell debris harbouring inclusion body was collected.

### Acetic acid extraction after Triton X-114 double washing of inclusion body

The isolated inclusion body was treated with Triton X-114 washing twice. It was suspended in Triton X-114 washing buffer (1% (v/v) Triton X-114 (Sigma), 1 mM EDTA, and 50 mM Tris–HCl; pH 8.0) with 10 mmol phenylmethanesulfonyl fluoride (PMSF; Sigma) and 1 mg/mL lysozyme (Bio Basic Canada Inc., Ontario, Canada), and agitated overnight. After centrifugation, inclusion bodies were resuspended in Triton X-114 washing buffer to remove PMSF and lysozyme. The isolated inclusion bodies were resuspended in 40 mL of 25% (v/v) acetic acid per gram wet weight to specifically extract recombinant fp-151. The extracted solution was centrifuged at 9,000 × g for 20 min at 4°C, and the supernatant was collected, dialyzed in distilled water (DW) with 0.1% (v/v) acetic acid, and freeze-dried.

### Ion exchange chromatography purification

For further purification of recombinant fp-151, fast protein liquid chromatography (FPLC; GE Healthcare, Buckinghamshire, UK) system was used. Sample was prepared by dissolving freeze-dried acid-extracted recombinant fp-151 in binding buffer (20 mM sodium acetate; pH 4.0). Then, cationic exchange column (HiTrap SP XL; GE Healthcare) was used for separating fp-151 and impurities through linear gradient of elution buffer (20 mM sodium acetate and 2 M NaCl; pH 4.0) with flow rate of 1 mL/min.

### Total protein quantification and SDS-PAGE analysis

Protein recovery of sample at each purification step was quantified by Bradford assay (Bio-Rad, Hercules, CA, USA) and 12% (w/v) SDS-PAGE. Band intensity on SDS-PAGE was analyzed by Gel-Pro Analyzer (Media Cybernetics, Rockville, MD, USA).

### LAL assay for endotoxin level determination

After setting the sample quantification to 1 mg/mL, endotoxin levels were determined using the Pierce® LAL Chromogenic Endotoxin Quantitation Kit (Thermo Scientific, Waltham, Massachusetts, USA), which is a quantitative endpoint assay for detection of Gram-negative bacterial endotoxins. The activation of a pro-enzyme in the modified LAL is catalyzed by bacterial endotoxin. A total 50 μL of LAL solution was added to each well of 50 μL sample or standard, and the plates were further incubated for 10 min at 37°C. Then, 100 μL substrate solution was added with 6 min incubation at 37°C followed by addition of 50 μL of 25% (v/v) acetic acid to stop reaction. The absorbance was measured at 410 nm using a microplate absorbance spectrophotometer (Bio-Rad).

### Total carbohydrate and lipid amount determination

The phenol-sulfuric acid assay was performed to determine the total quantity of carbohydrates in the samples at each purification step. All samples were freeze-dried and ten milligram of each sample was dissolved in 500 μL of 4% (w/v) phenol (Sigma) and 2.5 mL of 96% (v/v) sulfuric acid (Sigma). 1 mg/mL glucose was used in 5–50 μL of DW for the standard curve, and the optical density was measured at 490 nm. The total quantity of lipids in the samples at each step was analyzed by the sulfo-phospho vanillin colorimetric method. 10 mg of each sample (freeze-dried) was dissolved in chloroform. For the standard curve, 10–100 μg triolein were used. After drying of the chloroform, 100 μL DW was added. The samples were mixed with 2 mL of 96% (v/v) sulfuric acid, boiled for 10 min in a water bath and cooled for 5 min. The phosphoric acid-vanillin reagent was prepared with 85% (v/v) phosphoric acid (Sigma) and 1.2 g/L vanillin (Sigma). After treatment of 5 mL phosphoric acid-vanillin reagent, the samples were warmed at 37°C for 15 min and cooled for 10 min. After the reaction, the value of optical density was measured at 530 nm.

### HPLC analysis for protein purity determination

Recombinant fp-151 samples collected before and after ion exchange chromatography were analyzed by HPLC (Gilson, Middleton, WI, USA) with reverse phase Hypersil™ BDS 3 μm C18 column (4.6 × 100 mm; Thermo Scientific). Samples were eluted using a linear gradient of acetonitrile (0–100%, v/v) and HPLC grade water with 0.1% trifluoroacetic acid, and monitored by a UV detector at 280 nm.

### *In vitro* macrophage cell assays

To check inflammatory response of purified recombinant fp-151, expressions of inflammatory cytokines such as TNF-α and IL-6 were measured after treatment of the samples at each purification step on RAW 264.7 mouse macrophage cells. Total 3 × 10^4^ cells were seeded onto 96-well plate and incubated in Dulbecco’s modified Essential Medium (DMEM; Hyclone, Logan, UT, USA) supplemented with 10% (v/v) fetal bovine serum (Hyclone) and penicillin (100 unit/mL; Hyclone)/streptomycin (100 μg/mL; Hyclone) at 37°C under 5% CO_2_ humid atmosphere. After change of culture media, 4 μg MAP sample was added to the cells in a well and the secretion of inflammatory cytokines was analysed. Detection of expressed TNF-α and IL-6 in culture media was performed with sandwich enzyme-linked immunosorbent assay (ELISA). Capture and detection antibodies for TNF-α and IL-6 and standard recombinant mouse TNF-α and IL-6 were purchased from R&D Systems (Minneapolis, USA). All procedures were following manufacturer’s protocol.

Cell viability, cytotoxicity, and apoptosis were also examined using RAW 264.7 mouse macrophage cells. Total 2 × 10^4^ cells were plated onto 96-well plate and sample treatment was the same than the inflammation assay. Measurements of viability, cytotoxicity, and apoptosis of the cells were performed by ApoTox-Glo™ Triplex Assay (Promega, Madison, WI, USA) as following manufacturer’s protocol.

## Abbreviations

MAP: Mussel adhesive protein; LPS: Lipopolysaccharide; HPLC: High performance liquid chromatography; TNF-α: Tumor necrosis factor-alpha; IL-6: Interleukin-6; ELISA: Enzyme-linked immunosorbent assay; LAL: Limulus amoebocyte lysate; WC: Whole cells; IB: Isolated inclusion body containing MAP after disruption of CaCl_2_/EDTA-treated whole cells; X-114/114: Acetic acid-extracted MAP after inclusion body washing with Triton X-114 twice; IE: MAP fraction through cation exchange chromatography of acetic acid-extracted solution; ppMAP: purified MAP based on previous method.

## Competing interests

The authors declare no conflict of interest.

## Authors’ contributions

Conception and design of the study: JHS, BHC, YKJ and HC. Acquisition of data: YKJ, HC, SYB and BHC. Analysis and interpretation of data: BHC, YKJ and HC. Drafting the article: HC, BHC and YKJ. Revising it critically for important intellectual content: BHC, YKJ and HC. Final approval of the version to be submitted: all co-authors. All authors read and approved the final manuscript.
